# Collagen type IV alpha 1 chain (COL4A1) expression in the developing human lung

**DOI:** 10.1186/s12890-024-02875-4

**Published:** 2024-02-08

**Authors:** Laszlo Markasz, Hamid Mobini-Far, Richard Sindelar

**Affiliations:** 1 Department of Women’s and Children’s Health, Uppsala University, Uppsala, SE-751 85 Sweden; 2 Department of Pathology, Uppsala University Hospital, Uppsala, Sweden

**Keywords:** Basement membrane, COL4A1, Lung development, Digital image analysis, Cluster analysis

## Abstract

**Background:**

Collagen type IV alpha 1 chain (COL4A1) in the basement membrane is an important component during lung development, as suggested from animal models where COL4A1 has been shown to regulate alveolarization and angiogenesis. Less is known about its role in human lung development. Our aim was to study COL4A1 expression in preterm infants with different lung maturational and clinical features.

**Methods:**

COL4A1 expression in 115 lung samples from newborn infants (21-41 weeks’ gestational age; 0-228 days’ postnatal age [PNA]) was studied by immunohistochemistry combined with digital image analysis. Cluster analysis was performed to find subgroups according to immunohistologic and clinical data.

**Results:**

Patients were automatically categorized into 4 Groups depending on their COL4A1 expression. Expression of COL4A1 was mainly extracellular in Group 1, low in Group 2, intracellular in Group 3, and both extra- and intracellular in Group 4. Intracellular/extracellular ratio of COL4A1 expression related to PNA showed a distinctive postnatal maturational pattern on days 1-7, where intracellular expression of COL4A1 was overrepresented in extremely preterm infants.

**Conclusions:**

COL4A1 expression seems to be highly dynamic during the postnatal life due to a possible rapid remodeling of the basement membrane. Intracellular accumulation of COL4A1 in the lungs of extremely premature infants occurs more frequently between 1 and 7 postnatal days than during the first 24 hours. In view of the lung arrest described in extremely preterm infants, the pathological and/or developmental role of postnatally increased intracellular COL4A1 as marker for basement membrane turnover, needs to be further investigated.

**Supplementary Information:**

The online version contains supplementary material available at 10.1186/s12890-024-02875-4.

## Introduction

The basement membrane (BM) is an important component of the extracellular matrix (ECM) and provides a communication platform between epithelial and mesenchymal cells, thereby promoting proliferation and differentiation of the epithelium [1]. The interaction between epithelium and mesenchyme is necessary for normal lung development [2, 3], and involves a complex signaling network of peptide growth factors, transcriptional regulators and ECM proteins and their receptors [4].

BM components such as laminin, fibronectin and collagen type IV have been found to stimulate the migration of bovine bronchial epithelial cells in vitro [5]. It has been suggested that the composition of ECM at sites of epithelial injury may play an important role in the repair processes that occur after injury to the bronchial epithelium [5], by affecting both cell shape and the expression of surfactant protein genes in type II pneumocytes in vitro [6]. The main component of the epithelial BM is collagen IV and it is responsible for its strength [7]. During early development, laminin is sufficient for BM-like matrices, but at later embryonal stages collagen IV dominates as the stabilizing component of BM structures [7].

Both epithelial cells and fibroblasts produce collagen IV during lung development [8]. However, higher levels of collagen IV are observed in fibroblasts during the earlier stages of the development [8], whereas immature type II lung epithelial cells express collagen IV before the type II cell phenotype is completely defined [9]. There are six subunit chains of collagen IV, COL4A1–COL4A6, also named as α1–α6 chains [10, 11]. These six different α-chains are assembled only in three trimeric combinations, α1α1α2, α3α4α5, and α5α5α6 [1]. During lung maturation, the initial network of collagen IV is composed of α1α1α2 trimers which are partially replaced by the other two combinations of trimers in a tissue-specific manner with specialized BM structures and functions [7, 12], α1α1α2 trimers continuously exists along the subepithelial BM from the bronchi to alveoli, while α3α4α5 trimers appear only in BM of the alveoli [13]. Interestingly, bronchioalveolar stem cells (BASCs), which play an important role in both maintenance and repair of bronchiolar and alveolar cells [14], produce only α1α1α2 trimers [15].

Animal studies have shown that COL4A1 (α1 chain) plays a crucial role in both alveolarization and angiogenesis during lung development, especially during the saccular and alveolar phases [16–19]. The upregulation of COL4A1 gene has been localized to the lung interstitium and the developing alveolar septa, where proliferation, differentiation and migration of distal epithelial and myofibroblastic cells seem to be regulated by COL4A1 [16, 17]. The BM synthesis of collagen IV appears to be involved in angiogenesis [18], a process which is further promoted by NO [19].

Our knowledge on COL4A1 gene expression during lung development is mainly based on animal studies. Since the BM and its component collagen IV probably play a major role in the pathophysiological development of lung diseases in newborn infants [20–23] it is important to define COL4A1 expression in different lung conditions. The aim of the present study is to map COL4A1 expression in term and preterm newborn infants during different lung maturation stages and clinical features.

## Methods

### Lung tissue samples

Lung samples from each of the five lung lobes of 115 deceased newborn infants (born 1990-1996; postnatal age [PNA] 0–228 days) were obtained with informed written consent from the parents. The study was performed in accordance with relevant national guidelines and regulations. The study was approved by the Regional Ethical Review Board, Uppsala, Sweden (DNR 53/94; DNR 2019-03520). Autopsy was performed within 48 h of death. The same tissue collection has been described and investigated in three previous studies to determine the HA content [24] and RHAMM [25] and CD44 expression [26] of the lungs. Lung tissue from three adults were used as positive controls (informed written consent obtained before death). Adult samples were fully anonymized and no clinical data was available. The samples were stored in a 4% formaldehyde solution buffered by 10 g/l cetylpyridinium chloride to pH 7.3 until paraffin embedding. The paraffin embedding was done in a vacuum infiltration processor and included dehydration of the samples with graded alcohol series (70–99.5%) and clearing with xylene (100%). Embedded samples were sectioned (4 μm) by a microtome (HM355S, Microm, Germany) and mounted on slides.

### Patient data

**Fig. 1:**
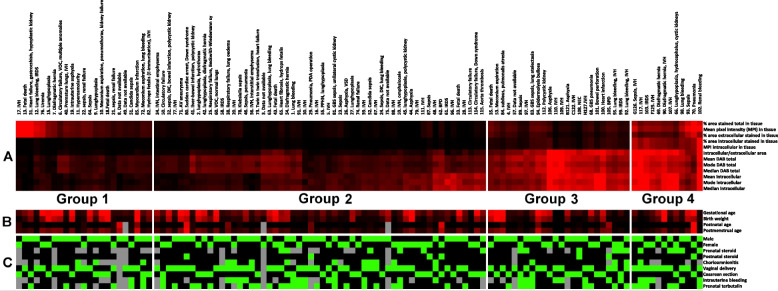
COL4A1 expression patterns after clustering and causes of death. **A** The pattern of parameters from the image analysis describes the distribution and the appearance of COL4A1 expression. Higher intensity in red corresponds to higher value. **B** Numerical clinical parameters (not included in the clustering). Higher intensity in red corresponds to higher value. **C** Non-numerical clinical parameters (not included in the clustering). Green = yes, black = no, grey = no data. Group 1: mainly *extracellular expression* of COL4A1 (% area extracellular stained in tissue elevated). Group 2: general *low expression* of COL4A1. Group 3: mainly *intracellular expression* of COL4A1 (intracellular/extracellular area ratio is elevated). Group 4: both *intracellular and extracellular expression* (% area extracellular stained in tissue, % area intracellular stained in tissue, intracellular/extracellular area are elevated)

Patient data were extracted from archived medical records. Of 115 infants, 43 were female (37%) and 72 male (63%); 96 infants were born preterm (83%) and 19 at term (17%). All pregnancies were evaluated by ultrasound examination at gestational weeks 14-17. Four infants died just before birth. The live born infants were treated at the Neonatal Intensive Care Unit, Uppsala University Children’s Hospital, Uppsala, Sweden. The patient characteristics are represented in Tables 1 and 2. The causes of death are listed in Fig. 1.

**Table 1:** Patients´ clinical characteristics

	Gestatonal age (weeks)	Birth weight (g)	Postnatal age at death	Postmenstrual age at death (weeks)
Minimum	21	365	7 hours	21
Maximum	41	4720	228 days	70
Mean	28,9	1512	15 days	31
Median	27	959	2 days	28

**Table 2:** Distribution of clinical parameters

**Gestational age (weeks)**	***n*** **= 116**
21-24	37
25-28	32
27-32	14
33-36	14
37-40	16
41-42	3
**Birth weight (g)**	***n*** **= 116**
365-1000	62
1001-1500	9
1501-2000	10
2001-3000	22
3001-4000	10
4001-4720	3
**Postnatal age at death (days)**	***n*** **= 116**
unknown	3
0-1	53
2-3	19
4-7	17
8-14	4
15-30	9
> 30	11
**Postmenstrual age at birth (weeks)**	***n*** **= 116**
21-24	15
24,1-28	42
28,1-32	14
32,1-36	13
36,1-40	14
> 44	8

### Immunohistochemistry

All samples were sectioned and stained on the same occasion for comparable analysis. Paraffin-embedded sections were deparaffinized through a graded series of xylol-ethanol. Lung sections were placed in pressure cooker (Biocare Medicals, USA) 125 °C for 4 minutes, followed by incubation with Target Retrieval Solution, Citrate pH 6 (S236984-2, Agilent, USA) for 30 minutes. To determine COL4A1 expression, immunohistochemistry was performed in Autostainer Link 48 (Agilent, USA) using EnVision FLEX visualization system (Agilent, USA) and counterstained with hematoxylin-eosin. Tissue sections were incubated 30 minutes at room temperature with primary antibody, a polyclonal rabbit anti-human COL4A1 antibody (1:300, SAB4500369 Sigma Aldrich®, USA). The accuracy of the staining pattern was confirmed with another polyclonal rabbit anti-human COL4A1 antibody PA5-85634 (1:300, Invitrogen®, USA) by including lung samples from eight neonates and using the same retrieval and staining procedures as mentioned above. Both antibodies were directed against the recombinant protein of human COL4A1. Positive control lung sections were used from three adults. Negative control sections were prepared by performing immunostaining procedures without adding primary antibodies. Stained sections were scanned by digital slide scanner (NanoZoomer S60, Hamamatsu, Japan) by using the same exposure times. Digitalized sections were examined by NDP.view2 (Hamamatsu, Japan) a whole slide viewing software. The same magnification (10 x objective) was used for all the images. Three representative areas per section/patient were exported into three images (size:23 MP, 6400 × 3616 pixels, type: RGB, format: TIFF). RGB image allowed the range of 255 intensity levels in the three color channels (red, green, blue), no saturated pixels could be observed.

### Software and image analysis

**Fig. 2:**
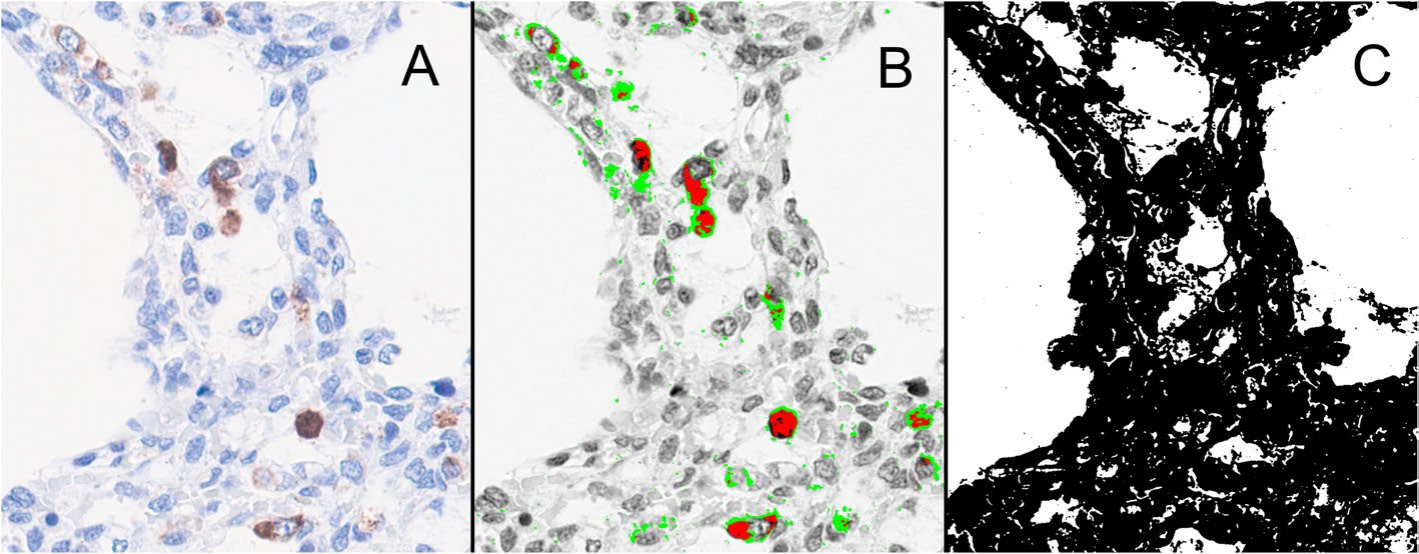
COL4A1 expression in the newborn lung (patient No.69). **A** DAB staining. **B** DAB staining after digital image processing. *Green:* low threshold area without high threshold area (mainly extracellular expression). *Red:* high threshold area alone (mainly intracellular expression). The green and red area together corresponds to the total COL4A1 expression. **C** The size of the tissue covered area (*black*)

A total of 357 images were sorted into stack and saved in TIFF format. FIJI [27] was used for semi-automatic image analysis. The color detection of DAB staining was performed by the IHC Toolbox plugin in FIJI, which is used to analyze samples stained by immunohistochemistry [25]. Working with image stacks during the evaluation process allowed for making the analysis comparable between images. After color detection of DAB staining, RGB color images were converted to 8-bit files. Inversion of the pixel intensity values resulted in higher pixel intensity corresponding to higher COL4A1 expression [25]. The result of DAB detection is presented in Fig. 2A-B. The size of the tissue covered area was measured by selecting a threshold level which separated the tissue from the background in the original RGB image (Fig. 2C). Before analysis, the same threshold window was set on all images in order to filter too low non-specific pixel values.

### COL4A1 expression

Three representative images per section were selected and used for the measurement of COL4A1 expression. A low threshold was set to determine the total COL4A1 expression in the whole tissue, and a high threshold to select high color density which mainly corresponded to the intracellular staining of COL4A1 (Fig. 2B). After the relevant threshold values were set for the whole project, the analysis was performed by using the Analyze Particles tool in FIJI. We chose 12 parameters to describe the characteristics of the digital images:

- Mean DAB total: mean value of pixel intensity level within the low threshold area, allowed comparing COL4A1 expression level between patients even if the area with COL4A1 expression or the tissue-covered area of the section was different
- Mode DAB total: most frequently occurring pixel intensity value within the low threshold area, corresponded to the highest peak in the histogram
- Median DAB total: median value of pixel intensity level within the low threshold area
- % area stained in total tissue: low threshold area / tissue covered area
- Mean intracellular: mean value of pixel intensity level within the high threshold area
- Mode intracellular: mode value of pixel intensity level within the high threshold area
- Median intracellular: median value of pixel intensity level within the high threshold area
- % area intracellular stained in tissue: high threshold area /tissue covered area
- % area stained in tissue: (low threshold area -high threshold area)/tissue covered area
- Mean pixel intensity in tissue: mean value of pixel intensity level with low threshold within the tissue covered area
- Mean pixel intensity intracellular in tissue: mean value of pixel intensity level with high threshold within the tissue covered area
- Intracellular / extracellular area: high threshold area/ (low threshold area -high threshold area)

Each parameter was estimated for the individual images. The mean values of three individual measurements for each patient were used for further evaluation.

### Cluster analysis

All the 12 parameters corresponding to COL4A1 expression were analyzed together by two-dimensional hierarchical clustering (Cluster 3.0 freeware) as previously described [25, 26]. The clustering algorithm sorted patients into groups depending on the relations of their parameters. The results were visualized by Java Treeview [28] as a map of color pixels. Higher intensity in red corresponded to higher value of a certain parameter.

### Statistical analysis

Two-sided Student’s *t*-test was performed to assess significant differences. For multiple comparisons, Bonferroni correction was used after one-way analysis of variance (ANOVA). Pearson’s correlation was used to show whether and how strongly pairs of variables were related.

## Results

### Clustering of patients

**Fig. 3:**
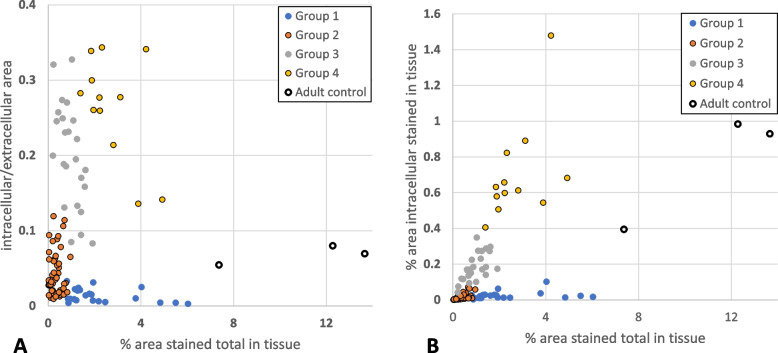
The distribution of intra- and extracellular expression of COL4A1 in Group 1-4 and adult controls. Intracellular/extracellular stained area ratio, % area stained total in tissue, and % area intracellular stained in tissue were selected to visualize the separation of Group 1-4. **A** shows intensity levels and **B** area of intracellular staining. Group 1: mainly *extracellular expression* of COL4A1 (% area stained in tissue is low). Group 2: general *low expression* of COL4A1. Group 3: mainly *intracellular expression* of COL4A1 (high intracellular/extracellular area ratio). Group 4: both *intracellular and extracellular expression* occurs (all the parameters are increased). Adult controls had larger total stained area than all infants

**Fig. 4:**
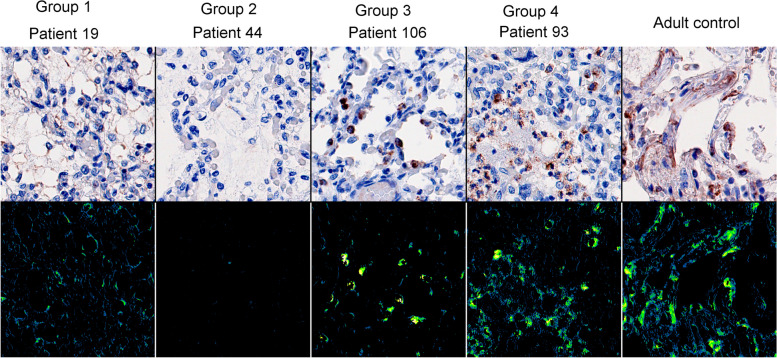
Representative microscopic images of the four groups and adult controls. Group 1: mainly *extracellular expression* of COL4A1. Group 2: general *low expression* of COL4A1. Group 3: mainly *intracellular expression* of COL4A1. Group 4: both *intracellular and extracellular* expression occurs (all parameters increase)

After the image analysis of the staining with antibody SAB4500369 (Sigma Aldrich®, USA), the clustering program sorted 1 hundred and 15 patients into 4 groups (Fig. 1). Each group was unique and showed an individual visual pattern of red pixels. The manual microscopic evaluation of COL4A1 staining with SAB4500369 (Sigma Aldrich®, USA) showed that COL4A1 expression appeared both intra- and extracellularly in the lung sections with different proportions between the patients. The antibody PA5-85634 (Invitrogen®, USA) stained the lung sections extracellularly exclusively, but with the same extracellular pattern and extracellular intensity levels as observed with SAB4500369 (Sigma Aldrich®, USA) (see in Supplement). This confirmed not only the accuracy of the staining with SAB4500369 (Sigma Aldrich®, USA) but also suggested indirectly that the intracellular appearance with this antibody is due to its specificity even against altered conformations of COL4A1 and not to artifacts or technical failures. Analyzing the staining with SAB4500369 (Sigma Aldrich®, USA), three parameters (% area stained total in tissue, % area intracellular stained in tissue, intracellular/extracellular stained area) showed the strongest correlation both with parameters describing stained area and staining intensity (Table 3). Figure 3 shows how the four groups were separated according to these three parameters. The pattern of intra- and extracellular staining is visualized in Fig. 4 by showing representative microscopic images of the four groups and an adult control: Group 1 (*n* = 23) represented patients with mainly extracellular expression of COL4A1; Group 2 (*n* = 56) showed general low COL4A1; Group 3 (*n* = 24) expressed COL4A1 mainly intracellularly; and Group 4 (*n* = 12) expressed both intracellular and extracellular expression (Figs. 1, 3 and 4). Adult controls had larger total stained area than infants (Fig. 3A-B). However, the ratio of intracellular to extracellular area in adults was low, similar to Group 1 and 2 (Fig. 3A). A larger total stained area corresponded to larger high-density area (intracellular staining) in adult controls, Group 3 and Group 4 but not in Group 1 and 2 (Fig. 3B).

**Table 3:**
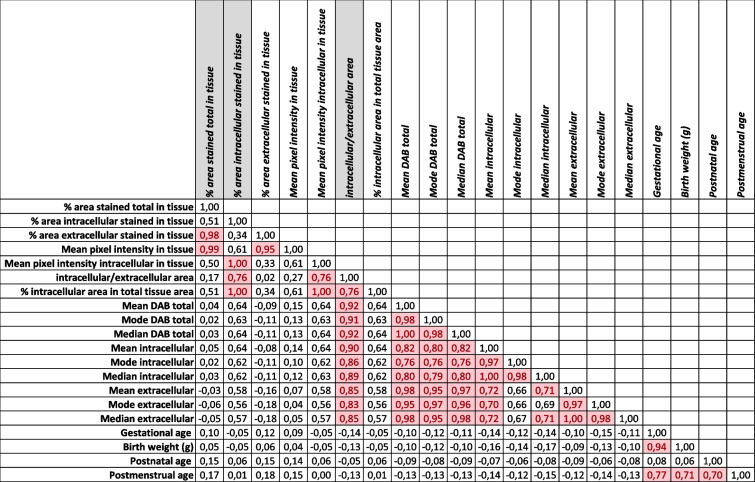
The correlation between parameters describing COL4A1 expression and clinical parameters. Correlation > 0.7 is marked with red. % area stained total in tissue, % area intracellular stained in tissue, and intracellular/extracellular stained area (marked with grey) showed the strongest correlation both with parameters describing stained area and staining intensity (parameters with mean, mode and median)

### Patients´ clinical characteristics in the groups

**Fig. 5:**
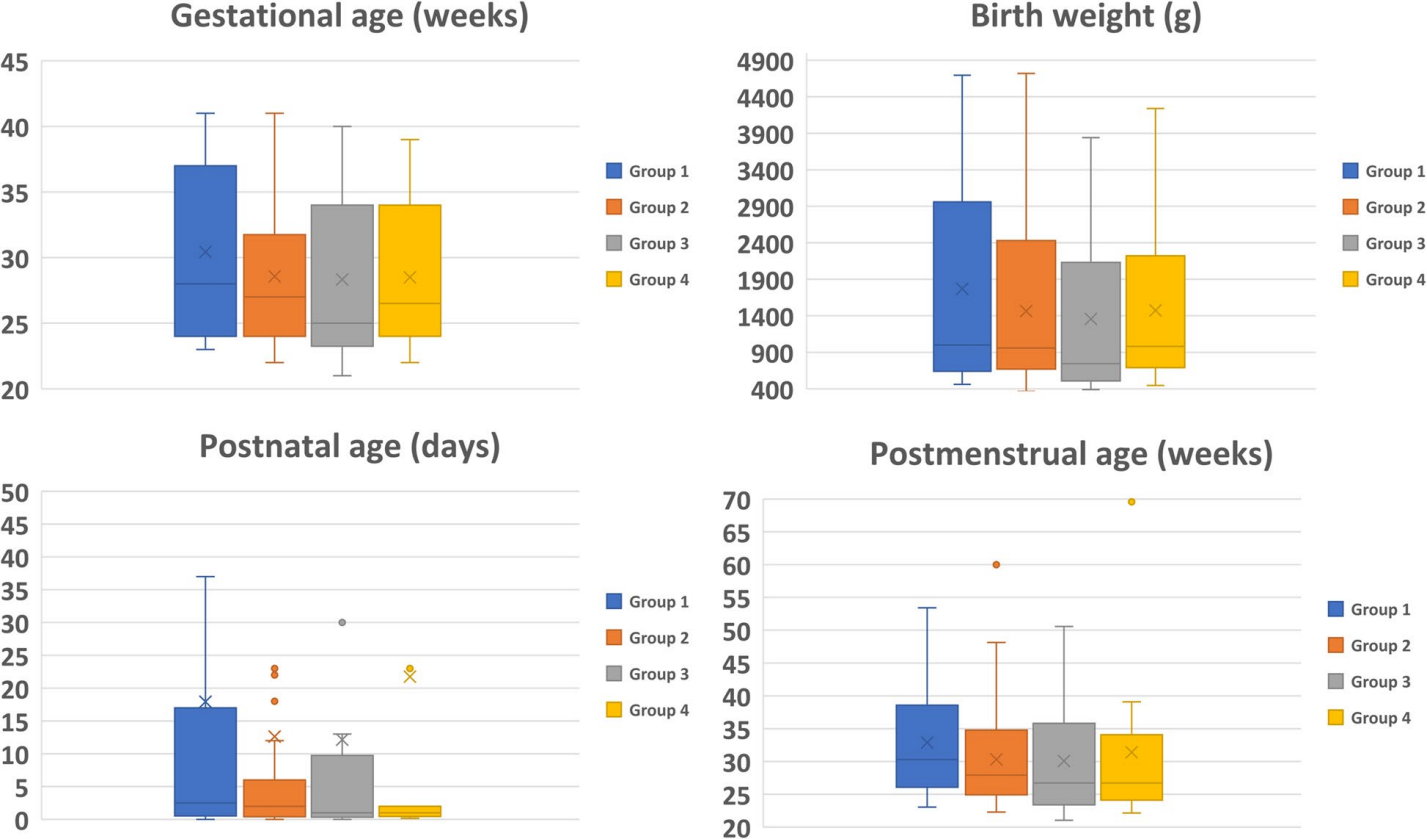
Patients´ clinical characteristics in Group 1-4. The groups did not differ statistically (data not shown) in gestational age, birth weight, postnatal age or postmenstrual age at death

The groups did not differ in gestational age (GA), birth weight (BW), PNA and postmenstrual age (PMA) at death (Figs. 1 and 5, statistic not shown). There were no differences between the groups in the proportion of males and females, perinatal complications, prenatal treatments or postnatal steroids (Fig. 1, statistic not shown). All groups were heterogeneous as to their cause of death (Fig. 1). As there were too few patients for each diagnosis in each group, statistical analysis was not feasible.

### The impact of PNA on COL4A1 expression

**Fig. 6:**
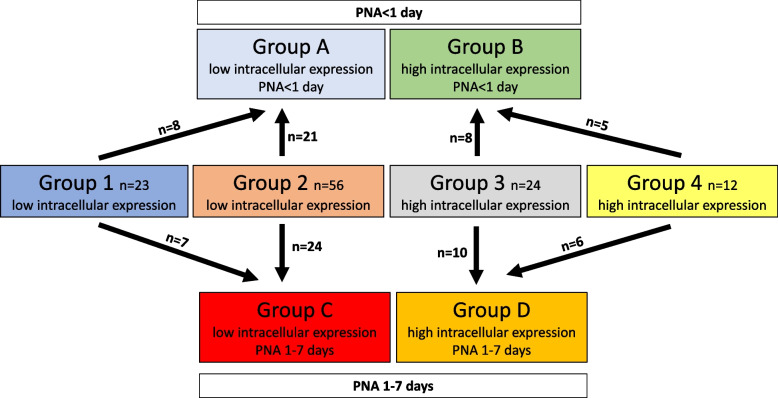
Sorting of patients from Group 1-4 into Group A-D according to postnatal age at death < 1 day vs 1-7 days

**Fig. 7:**
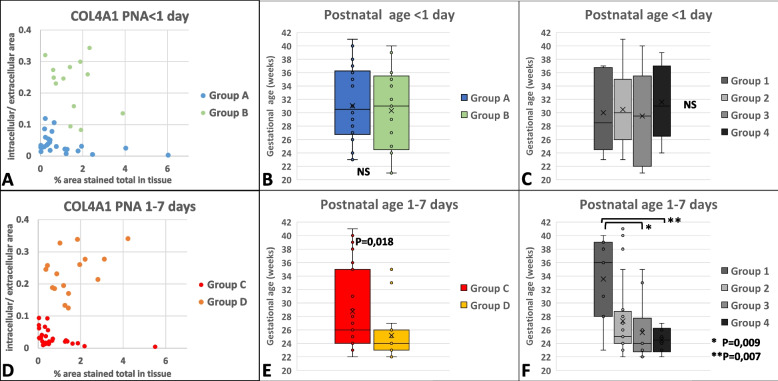
The distribution of intra- and extracellular expression of COL4A1 according to postnatal age at death. **A**, **B**, **C**: postnatal age at death < 1 day. **D**, **E**, **F**: postnatal age at death 1-7 days. **B**, **E** Distribution of GA in the groups. No difference between Group A and Group B; lower GA in Group D compared to Group C. C, F: Distribution of patients according to GA in the original groups

PNA did not correlate with any of the COL4A1 expression parameters in general (Table 3). Patients were further sorted into groups according to their PNA at death (Fig. 6), in order to uncover associations between COL4A1 expression patterns and pre- and postnatal factors. PNA at death < 1 d was considered within limits for this differentiation. Forty-two patients (36.5%) died within 1 day, 47 patients (40,8%) between 1 and 7 days, and 26 patients after 1 week. Group 1 was sorted together with Group 2, and Group 3 was sorted together with Group 4, since they shared the same phenotype with low or high intracellular COL4A1 expression respectively. Patients with PNA < 1 day from Group 1 and Group 2 were sorted into Group A, and from Group 3 and Group 4 they were sorted into Group B (Fig. 6, Fig. 7A). Patients with PNA 1-7 days from Group 1 and Group 2 were sorted into Group C, and from Group 3 and Group 4 they were sorted into Group D (Fig. 6, Fig. 7D). There was no difference in GA between Group A (mean GA: 30.30 weeks) and Group B (mean GA: 30.34 weeks) (Fig. 6 and Fig. 7B). However, infants in Group D had lower GA (mean GA 25.2 weeks) compared to Group C (mean GA: 28.74 weeks) as showed in Fig. 7E. Fig. 7C and F show the distribution of patients according to GA in the original groups (Group 1-4) with PNA < 1 day or with PNA 1-7 days. Patients with PNA < 1 day exhibited similar GA in all the four groups (Fig. 7C); whereas patients with PNA 1-7 days had lower GA in Group 3 and 4 (high intracellular expression) compared to Group 1 (mainly extracellular expression). Differences between Group 2 (general low expression) and the other groups were statistically not significant (Fig. 7F). Thus, the results presented in Fig. 7B and E may indicate that after 1 day PNA, intracellular expression of COL4A1 is overrepresented among extremely preterm infants with an earlier stage of lung development.

Patients with PNA at death longer than 7 days were excluded from this part of the analysis due to the low number of infants, the wide-spread time range (8-228 days, *n* = 26), the possible extensive postnatal remodeling of the lung structure and the increasing number of other postnatal factors.

## Discussion

Animal models suggest that COL4A1 may have an important role in lung development. Human studies are difficult to perform due to ethical limitations. To our knowledge this is the first study describing COL4A1 expression pattern in the developing human lung. In order to distinguish between possible prenatal and postnatal factors that might affect the regulation of COL4A1 synthesis and BM formation, COL4A1 expression was investigated in infants who died during their first day of life and on day 1-7. A new finding was the observation of intracellular accumulation of COL4A1 in many of patients and specifically in extremely preterm infants on day 1-7. Since the present study lacks controls and all patients died in severe organ failure and/or clinical complications, our results allow only for cautious interpretations. However, animal studies and the clinical manifestation of mutations in COL4A1 were helpful to make some indirect conclusions.

Heterozygous mouse mutants with deletion of exon 41 in the collagen IV gene results in increased intracellular accumulation and decreased extracellular collagen, which might contribute to the reported abnormal angiogenesis [29]. Mutations in humans can also lead to decreased extracellular COL4A1 which contributes to sporadic cerebrovascular diseases and intracranial hemorrhage [21]. Increased intracellular accumulation of COL4A1 has not been described in physiological conditions. We observed that increased intracellular accumulation of COL4A1 in the lung occurs with higher frequency in extremely premature infants than in term infants after the first day of life. This phenomenon may indicate that intrauterine mechanisms are not alone in this process but the birth itself or other postnatal factors may act as main catalysts together with prematurity.

Intracellular accumulation can be caused by increased intracellular biosynthesis or inhibited secretion of COL4A1, which seems to be associated with both GA and PNA in our patients and may also determine BM formation. Collagen IV scaffolds are synthesized both by intracellular and extracellular mechanisms. One COL4A2 and two COL4A1 peptides assemble into heterotrimers (α1α1α2) within the endoplasmic reticulum (ER) before being transported to the Golgi and secreted into the extracellular space. This process needs several post-translational modifications where heterotrimers are packaged into vesicles for secretion into the ECM [30]. Further protomer assembly into a three-dimensional scaffold occurs extracellularly [31]. Intracellular and extracellular collagen IV-associated chaperones and chaperone-like proteins are critical to ensure proper secretion and stereotypic assembly of collagen IV networks in BMs [32]. Mutation or disfunction of proteins involved in post-translational modifications (HSP47, SPARC) can result in intracellular accumulation and impaired excretion of collagen IV [20]. It has been shown that accumulation of α1α1α2 heterotrimers within cells leads to an activation of ER stress resulting in unfolded protein response (UPS) with cytotoxic effects and apoptosis [33, 34]. However, the elimination of accumulated collagen by ER-associated degradation or autophagy counteract this process and promotes cell survival [35]. According to our results, it may be reasonable to assume that similar mechanisms lead to intracellular accumulation of COL4A1 and disturbed postnatal lung development in extremely preterm infants. We speculate that intracellular accumulation of COL4A1 after the first postnatal day might be a sign of perinatal processes involved in postnatal lung development and/or healing after lung injury in extremely preterm infants. Since the antibody SAB4500369 (Sigma Aldrich®, USA) was produced against synthetic COL4A1 peptide residues and seems to show specificity even against altered conformation of COL4A1, we have to take into account the possibility that it labels both folded and unfolded COL4A1 chains. In that case, increased intracellular staining might be a sign of unfolded protein accumulation that may lead to UPS.

Fujimoto et al. found that mRNA expression of COL4A1 was increased by 24 hours glucocorticoid treatment in cultured porcine trabecular meshwork cells [36]. The effect of antenatal steroid treatment on COL4A1 expression in the preterm lung cannot be ruled out. Although our study included patients treated with steroids, the low number of treatments [antenatal: *n* = 17 (14.8%), postnatal: *n* = 7 (6.1%)] combined with a wide range of postnatal ages (Fig. 1) did not allow for a statistical analysis of this possible effect.

Interestingly, many patients in our study (Group 2) had low extracellular COL4A1 expression without association with lung maturation level or type of lung disease. Several studies confirmed that the BM undergoes continuous remodeling in the lung. Grant et al. showed enhanced remodeling of BM in the saccular phase of rat lung development resulting discontinuities of BM exclusively under type II pneumocytes in association with their differentiation [37]. These discontinuities in the BM enables epithelial-mesenchymal cell to cell contacts during type II pneumocyte maturation. To be noticed, these events occur earlier in female rats showing similarities to the gender associated differences observed in preterm infants related to survival and outcomes [38, 39]. COL4A1 as a main component of BM might have an important role in this process. Several authors found that COL4A1 expression increased during the saccular and alveolar stage in the lung [8, 16, 17]. On the other hand, it was also shown that the degradation of collagen IV can occur rapidly during the late fetal lung development even in physiological circumstances [40]. These contradictory findings can partly be explained by the assumption that there exists a dynamic remodeling of BM. Differences between high and low COL4A1 expression levels among our patients may depend on a similar remodeling which possibly comprises both production or degradation processes.

The wide range of COL4A1 levels without association to GA or PNA in our study may indicate that postnatal COL4A1 expression changes are highly dynamic due to rapid remodeling in the ECM. Matrix metalloproteinases (MMPs) are main actors in the remodeling of BM and collagen IV degradation during embryonic development and morphogenesis. MMPs can be stimulated by hormones, growth factors, cytokines and inhibited by steroids and transforming growth factor beta (TGF-beta) [41]. Both fetal lung epithelial cells and fibroblasts express 72 and 92-kDa type IV collagenases (MMP-2 and MMP-9) which may play a role in the remodeling of BM in the lung [8]. Increased MMP activity is described in airway inflammation causing BM injury [42], which might have occurred even in the majority of our patients. MMP-2 as well as MMP-9 can proteolytically cleave latent TGF-beta and TGF-beta activation can remodel tissue structures [43]. Low MMP-2 level at birth have been associate with the development of bronchopulmonary dysplasia (BPD) [44]. This is concordance with the findings from Aghai et al. where patients eventually diagnosed with BPD displayed lower serum levels of collagen IV fragments compared to controls already during their first week of life [45]. The same study found two other valuable observations: (1) individuals with the lowest collagen IV values during their first week of life required significantly longer neonatal intensive care than those with the highest values irrespective of gestational age; and (2) collagen IV fragment levels in the serum increased postnatally during the first month in all patients independent of outcome [45]. These observations suggest that BM degradation may differ depending on postnatal ages and the severity of lung disease. Comparisons are difficult between these studies and ours, as we studied tissue expression of COL4A1 and not serum levels, and as the majority of our patients deceased within 1 week. However, our observations are in line with these studies since all our patients suffered critical illness and many showed low COL4A1 expression at low PNA. Furthermore, the wide range of overall COL4A1 expression without association to PNA or PMA in our study, suggests that dynamic remodeling of BM do occur postnatally.

Studies based on animal models showed how impaired remodeling of the basement membrane can initiate fibroblast proliferation and fibrosis. An important source of fibroblasts in fibrosis is provided by epithelial-mesenchymal transition (EMT) where epithelial cells are phenotypically transformed into mesenchymal cells (fibroblasts) due to epithelial stress or inflammation [46]. EMT seems to be involved even in lung fibrosis resulting irreversible remodeling of the pulmonary tissue [47]. Inhibition of collagen IV assembly or proteolytic digestion of collagen IV facilitates EMT in vitro. Such changes in BM architecture can later potentially lead to up-regulation of TGF-beta 1, which contributes to EMT [48]. The transformed epithelial cells have the ability to secrete MMP-2 which continues specifically to degrade BM [49]. There is evidence that TGF-beta1 induces EMT even in human alveolar epithelial cells [50, 51]. The fibrosis inducing effect of TGF-beta in the lung was previously presented in a rat model [52]. Increased TGF-beta signaling and levels of TGF-beta ligands are associated with both experimental and clinical BPD [53, 54].

As a marker for remodeling of the pulmonary BM, our data suggest that impaired COL4A1 expression/distribution during the first postnatal week might contribute to later structural changes in the lungs of extremely preterm infants.

## Conclusions

As shown in our study, expression levels and intra-extracellular distribution of COL4A1 have a high variability in preterm neonates. After the first day of life, intracellular accumulation of COL4A1 occurs predominantly in extremely preterm infants. Prematurity in combination with PNA and other postnatal factors seem to overweigh over prenatal processes and to determine the level of expression and cellular distribution of COL4A1. We hypothesize that COL4A1 expression level is dynamic during the postnatal life due to rapid remodeling of BM. Increased intracellular COL4A1 expression after the first day of life might be pathological in neonates, as a sign of lung injury or repair, and occurs more frequently in extremely preterm infants. The cause of increased intracellular COL4A1 expression needs to be further investigated; however, the phenomenon may be linked to later pathological structural or functional changes in the lungs of extremely preterm infants and lends itself for possible future interventions and/or modulations.

### Supplementary Information

**Additional file 1: Supplement 1.** The staining pattern of representative sections with COL4A1 antibodies. Lung samples from two adults and four infants were stained with two polyclonal rabbit anti-human COL4A1 antibodies, 1:300: SAB4500369 (Sigma Aldrich, USA) and PA5-85634 (Invitrogen®, USA). Both antibodies are produced against recombinant peptide residues of human COL4A1. The four infants (31, 81, 106, 93) represented the four groups. Both antibodies stained lung sections from adults with a similar pattern. According to lung sections from infants, SAB4500369 stained both intracellular and extracellular sites; in Group 1 the staining appeared extracellularly, in Group 3 intracellularly and in Group 4 both intra- and extracellularly. Group 2 had a weak staining. PA5-85634 stained extracellular sites exclusively. The staining of the extracellular sites showed the same pattern and intensity levels in the respective patients for both antibodies.

## Data Availability

The datasets used and/or analysed during the current study are available from the corresponding author on reasonable request.

## Abbreviations

COL4A1: Collagen type IV alpha 1 chain
PNA: Postnatal age
BM: Basement membrane
ECM: Extracellular matrix
BASCs: Bronchioalveolar stem cells
GA: Gestational age
BW: Birth weigt
PMA: Postmenstrual age
MMPs: Matrix metalloproteinases
BPD: Bronchopulmonary dysplasia
TGF-beta: Transforming growth factor beta
EMT: Epithelial mesenchymal transition
