# Supplementary figures and images for: Collagen type IV alpha 1 chain (COL4A1) expression in the developing human lung
Source: BMC Pulm Med. 2024 Feb 8;24:75. doi: 10.1186/s12890-024-02875-4 (PMC10851591; doi:10.1186/s12890-024-02875-4)

## Slide 1
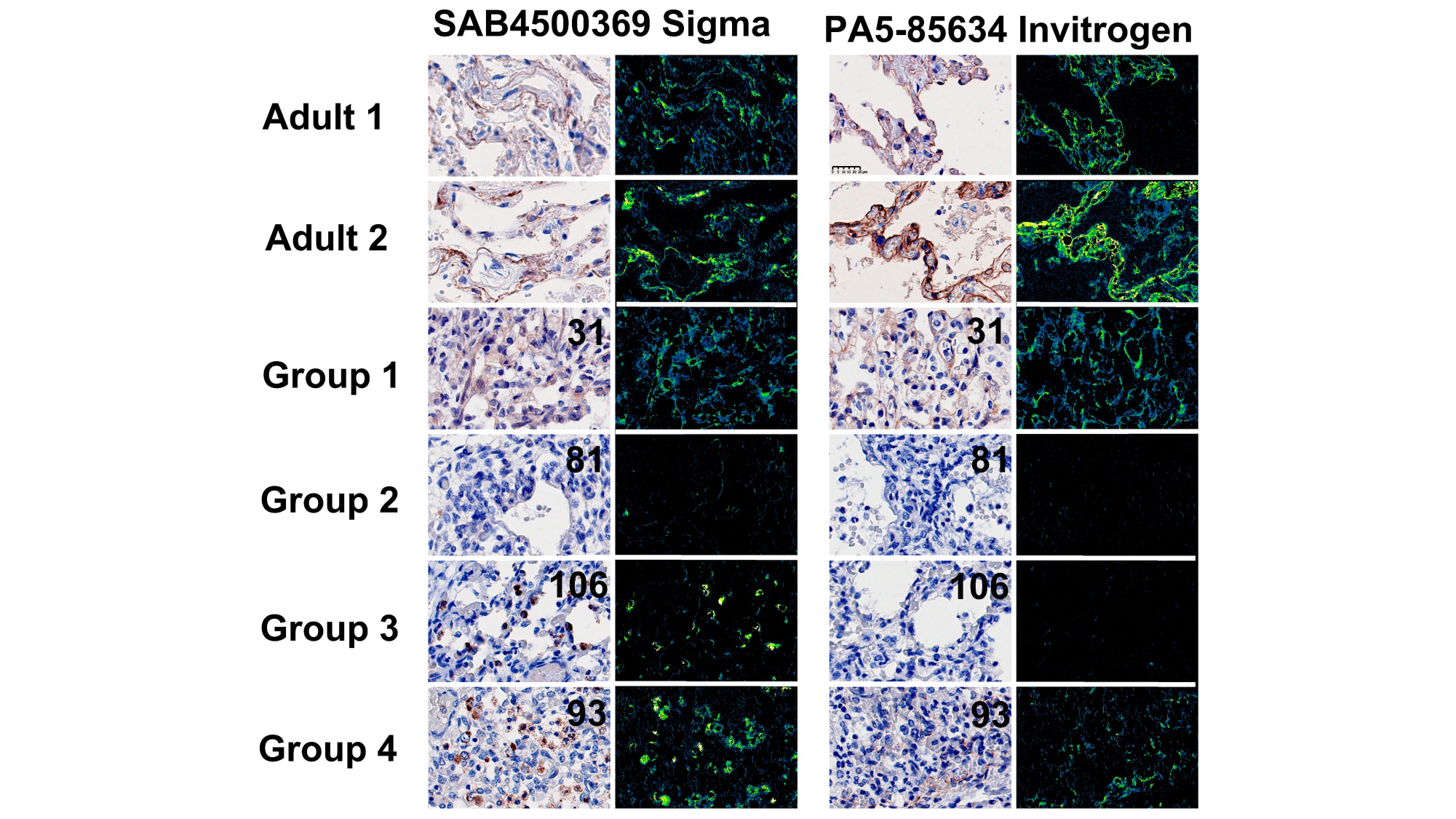

Supplement: Supplementary file 1 — Additional file 1: Supplement 1. The staining pattern of representative sections with COL4A1 antibodies. Lung samples from two adults and four infants were stained with two polyclonal rabbit anti-human COL4A1 antibodies, 1:300: SAB4500369 (Sigma Aldrich, USA) and PA5-85634 (Invitrogen®, USA). Both antibodies are produced against recombinant peptide residues of human COL4A1. The four infants (31, 81, 106, 93) represented the four groups. Both antibodies stained lung sections from adults with a similar pattern. According to lung sections from infants, SAB4500369 stained both intracellular and extracellular sites; in Group 1 the staining appeared extracellularly, in Group 3 intracellularly and in Group 4 both intra- and extracellularly. Group 2 had a weak staining. PA5-85634 stained extracellular sites exclusively. The staining of the extracellular sites showed the same pattern and intensity levels in the respective patients for both antibodies. [file 12890_2024_2875_MOESM1_ESM.pptx]
